# The Effects of Lithium on Proprioceptive Sensory Function and Nerve Conduction

**DOI:** 10.3390/neurosci4040023

**Published:** 2023-10-20

**Authors:** Kaitlyn E. Brock, Elizabeth R. Elliott, Alaina C. Taul, Artin Asadipooya, Devin Bocook, Tessa Burnette, Isha V. Chauhan, Bilal Chhadh, Ryan Crane, Ashley Glover, Joshua Griffith, JayLa A. Hudson, Hassan Kashif, Samuel O. Nwadialo, Devan M. Neely, Adel Nukic, Deep R. Patel, Gretchen L. Ruschman, Johnathan C. Sales, Terra Yarbrough, Robin L. Cooper

**Affiliations:** Department of Biology, University of Kentucky, Lexington, KY 40506-0225, USA

**Keywords:** conduction, crustacean, lithium, proprioception, recruitment, sensory

## Abstract

Animals are exposed to lithium (Li^+^) in the natural environment as well as by contact with industrial sources and therapeutic treatments. Low levels of exposure over time and high volumes of acute levels can be harmful and even toxic. The following study examines the effect of high-volume acute levels of Li^+^ on sensory nerve function and nerve conduction. A proprioceptive nerve in the limbs of a marine crab (*Callinectes sapidus*) was used as a model to address the effects on stretch-activated channels (SACs) and evoked nerve conduction. The substitution of Li^+^ for Na^+^ in the bathing saline slowed nerve conduction rapidly; however, several minutes were required before the SACs in sensory endings were affected. The evoked compound action potential slowed in conduction and slightly decreased in amplitude, while the frequency of nerve activity with joint movement and chordotonal organ stretching significantly decreased. Both altered responses could be partially restored with the return of a Na^+^-containing saline. Long-term exposure to Li^+^ may alter the function of SACs in organisms related to proprioception and nerve conduction, but it remains to be investigated.

## 1. Introduction

It is well known that the lithium ion (Li^+^) can substitute for the sodium ion (Na^+^) in the function of Na^+^ channels. Many cell types illustrate that cells can conduct electrical impulses with Li^+^ and that cells, particularly neurons, can still be electrically excited in the presence of Li^+^ or even with the full substitution of extracellular Na^+^ for Li^+^ [1]. This illustrates that Li^+^ does not impede Na^+^ channel conductance, potentially providing a similar electrical response; however, this replacement does not always result in the same electrophysiological responses as the original cell. Some cells even have regional differences in electrical response within the presence of Li^+^ as compared to Na^+^ [2].

Clinically, Li^+^ has been used to treat bipolar depression and, in some cases, epilepsy [3,4]. Due to the side effects of Li^+^ treatments, monitoring of kidney and thyroid function is necessary [4,5,6]. Many of lithium’s actions have been attributed to the effects of it altering cellular physiology. In rodent anterior pituitary cells, Li^+^ blocked K^+^ channels [7]. However, in rodent hippocampal neurons, it was shown that Li^+^ did not block the K+ channels directly and instead did so by lowering the Ca^2+^. This affected the neuronal excitability related to A-type K^+^ currents [8]. Additionally, the effects of altered efficacy in ion exchangers and pumps have been observed in various tissues [9,10,11,12]. Lithium nephrotoxicity is a concern for individuals being treated for bipolar depression via lithium treatments [13]. These lithium treatments have also been implicated in the dysfunction of the thyroid and parathyroid glands by yet unknown mechanisms [14]. There are differences in long-term (from hours to days) exposure to Li^+^ as compared to the immediate effects (from seconds to minutes), which may involve some compensatory or homeostatic regulation in the cells [15,16]. Within intact organisms, there can be changes in ionic balance, such as an increase in extracellular K^+^ ions with increased Li^+^. Slight changes in the concentration of extracellular K^+^ can have direct effects on the membrane potential and electrical excitability of cells [17,18,19]. It is assumed that the mechanisms of altered extracellular K^+^ may be related to the perturbation of the Na^+^-K^+^ ATPase pump [20].

There are still mechanisms of actions not understood with clinical Li^+^ treatments on many aspects of neuronal function, such as sensory modalities. For example, there are many types of stretch-activated channels (SACs) that function to monitor a myriad of physiological functions, including blood pressure, touch, pain, and proprioceptive function in animals, as well as pressure-sensitive channels (i.e., PIEZO) found in plants [21,22,23]. Many types of SACs are ionotropic. It has yet to be addressed how the Li^+^ ion may affect the flux and electrical responses of the cell. For example, the DEG/ENaCs (degenerin/epithelial sodium channels) known to be present in invertebrates and vertebrates exhibit Na^+^ flux as a major contributor to current [24].

Although there is likely no clinical reason to fully exchange Na^+^ for Li^+^, there remains theoretical interest in understanding the effects of such a substitution. Experimentation would allow for a better understanding of the consequences behind experimental applications of lithium in isolated tissues and cells. Investigating the biophysical responses to the substitution of Na^+^ with Li^+^ in various tissue and organism types allows for comparative addition to the knowledge of how different cells respond. Historically, invertebrate models have been used to address how cells respond to ionic perturbations and ionic properties of cells (especially neurons), with very impactful results. Particularly useful from an environmental standpoint is the ease by which many invertebrate neuronal types may be maintained (given the right temperature and minimal saline) for recording the electrical activity of individual neurons or nerves. Historically, crustaceans have provided experimental invertebrate models for addressing animal behavior, ion flux, transport, function of sensory neurons, synaptic transmission, and electrical conduction [25,26,27,28,29,30,31,32,33,34,35,36,37,38,39]. From the earliest studies of crustaceans, physiological research moved to incorporate insect, amphibian, and mammalian models.

The muscle receptor organ (MRO) of the crawfish has been a model of proprioceptive function for years, supporting a better understanding of how muscle spindles function in vertebrates [40,41,42,43,44,45]. As early as 1968, the effects of replacing extracellular Na^+^ with Li^+^ on neuronal function were addressed with the MRO preparation [2]. By performing single neuron recordings in the sensory cell body, it was shown that there was an initial enhanced excitability of the membrane with the production of spontaneous action potentials, which was then followed by a decrease in excitability. The neuronal cell bodies of the MRO are closely associated with the sensory endings. The axons did not show the same phenomena, resulting in various responses and indicating that regional differences exist along a single neuron. The reason behind observed mechanistic differences along a single neuron has yet to be fully described. Other useful invertebrate proprioceptive organ models are the chordotonal organs in the joints of insects and crustaceans [46,47]. In many readily accessible animal models like insects, the organs are small and delicate compared to the robust and larger chordotonal organs of crustacean limbs [48,49,50,51]. The crab chordotonal organs have been used to observe the anatomical structure of sensory endings, address mechanical–electrical coupling via SACs, and examine differences between subtypes of dynamic and static displacement-sensitive neurons [52,53,54,55,56,57]. Large crabs, such as the common Blue crab (*Callinectes sapidus*) or Dungeness crab (*Cancer magister*), feature a relatively long nerve (10 to 15 cm), which can be taken for observation from the most distal chordotonal organ of a limb to the base of the thorax. This renders the process of addressing alterations to electrical conduction in various types of bathing media easier [58].

The SACs in insect and crustacean chordotonal organs have yet to be fully described, either in terms of pharmacological or mechano-electrical transduction properties. The subtype present in crab chordotonal organs appears to maintain function without Ca^2+^ ions in the bathing environment [58]. The SACs are not altered by traditional agonists or antagonists like amiloride, ruthenium red, or streptomycin [59,60]; they are also not altered by selective compounds for PIEZO 1 subtype SACs (i.e., YODA 1, JEDI 2, OB 1, and DOOKU) [60]. Crab proprioceptive organs are being used as neuronal models for marine species to address the effects of heavy metal exposure, concepts of neurophysiology, and pharmacological profiling of SACs [61,62,63,64,65].

The purpose of this investigation was to examine the effects of ion substitution (Li^+^ for Na^+^) on a model sensory system and signal transmission along an isolated nerve.

## 2. Materials and Methods

The general procedures are similar to those previously described in detail [58,62] and in video format [66].

### 2.1. Animals

Blue crabs (Callinectes sapidus) were obtained from a local supermarket in Lexington, KY, USA, which had been delivered from a distribution center in Atlanta, GA, USA. They were bought and maintained in a seawater aquarium for several days prior to ensure that the organisms were in good health. Adult crabs of 10–15 cm carapace width (from point to point) were used, and only if they were active upon autotomizing a leg for experimentation.

### 2.2. Dissection and Physiology

**Figure 1:**
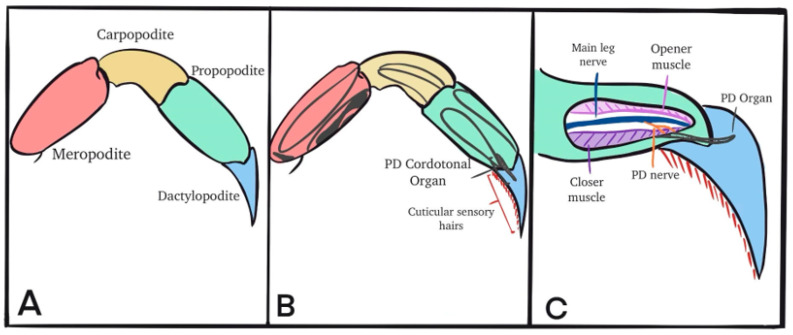
The isolation of the PD nerve for electrophysiological recordings. (**A**) The leg segments are shown, and the chordotonal organs are named by the joint they monitor. (**B**) The PD organ spans the most distal joint in the limb between the propodite and dactylopodite. (**C**) The PD nerve branches away from the main leg nerve near the base of the chordotonal strand.

Autotomization of the crab’s first or second walking leg was induced by lightly pinching the base of the leg with pliers. The propodite–dactylopodite (PD) chordotonal organ spans the last segment of the leg (Figure 1B) and was exposed by cutting a window of the cuticle on both sides of the leg (in the propodite segment; Figure 1C). With a window in the cuticle, the PD nerve can be observed independently of the main leg nerve (Figure 1B). The chordotonal organ spans the PD joint. After the windows were made and the cuticle removed, the leg was pinned in a Sylgard-lined dish and bathed in saline. The standard crab saline used during recordings of the sensory nerves consisted of (in mM) 470 NaCl, 7.9 KCl, 15.0 CaCl_2_·2H_2_O, 6.98 MgCl_2_·6H_2_O, 11.0 dextrose, 5 HEPES ((4-(2-hydroxyethyl)-1-piperazineethanesulfonic acid) acid, and 5 HEPES base adjusted to pH 7.5. To examine the effect of Li^+^, the NaCl was exchanged for LiCl at the same molar concentration.

**Figure 2:**
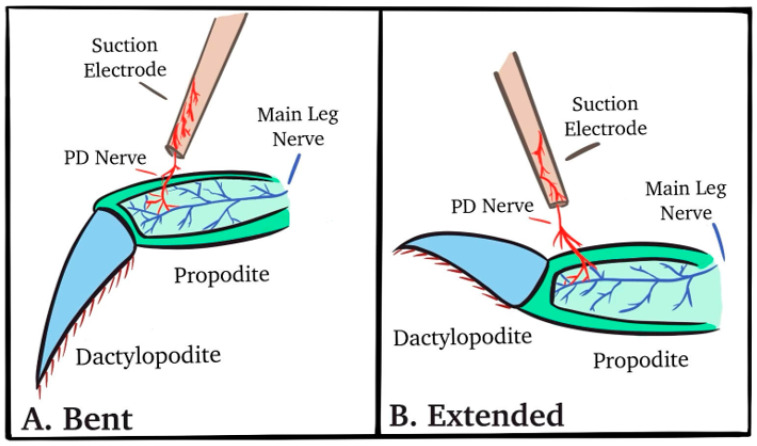
Exposing the PD nerve for recording nerve activity. (**A**) A length of PD nerve can be isolated from the main leg nerve and pulled into a suction electrode. The joint is bent (**A**) and extended (**B**) while nerve activity is recorded.

The PD nerve was left intact to record activity from dynamic and static position-sensitive neurons (Figure 2). The dactyl was moved from a bent (i.e., flexed) position to an extended (i.e., open) position within a one-second time frame, held for at least ten seconds, and then moved back to the starting position (Figure 2B). An insect dissecting pin stuck into the recording dish was used as a stop mark to ensure consistency in movement range among the trials.

**Figure 3:**
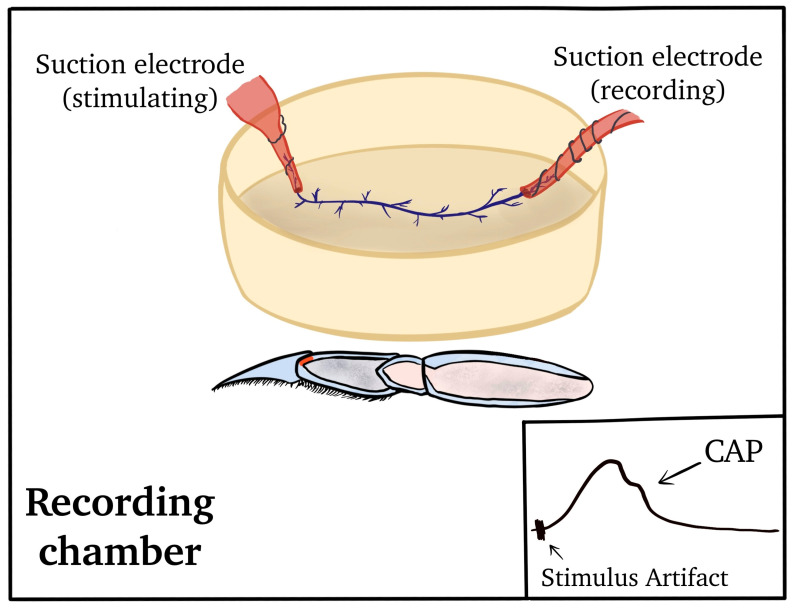
Set-up for recording activity of the PD nerve via compound action potentials (CAPs). The proximal end of the main leg nerve was used for the actual recording, while the distal end was used to provide stimulation to induce CAPs. In this arrangement, position-sensitive neurons are not firing since the sensory endings are removed.

Compound action potentials (CAPs) are initiated by stimulation at the proximal end of the PD nerve after isolation from the main leg nerve (Figure 3). The PD nerve was then cut away from the PD organ so that isolated CAPs could be recorded, both in normal bathing media and upon exposure to experimental solutions.

**Figure 4:**
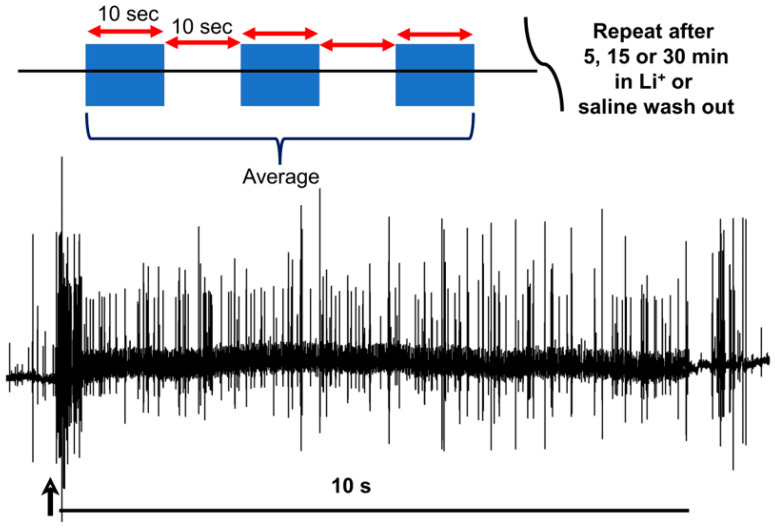
The experimental paradigm behind joint displacement and spike analysis. The joint is displaced from a flexed position to an extended one across a single second, held in place for at least ten more, and then moved back to flexed position. This was repeated three times in each bathing solution. An average of the activity across all three trials was used in conjunction with the raw data to assess the effects of changing the medium. The duration of exposure for each preparation depended on the medium in question. The spike count was obtained by counting the number of spikes from the beginning of the movement (across one second) through the next nine seconds of static positioning, and it was used as an index of PD organ neural activity. The arrow marks the beginning of the movement.

The numbers of extracellular recorded action potentials (i.e., spikes) recorded over the first ten seconds after joint displacement began were used as an index of neural activity. In each bathing condition, the joint was displaced thrice, with at least a ten-second pause between displacements. The number of spikes in each of the three trials was averaged, allowing for both graphical representation and for drawing comparisons among bathing conditions (Figure 4).

### 2.3. Statistical Analysis

Paired *t*-tests were used to examine differences in response before and after solution exchange, while normality was established using the Shapiro–Wilk test. The Wilcoxon rank sum non-parametric test was used when appropriate. The analysis was performed with Sigma Stat software. A *p*-value of <0.05 was considered statistically significant.

## 3. Results

**Figure 5:**
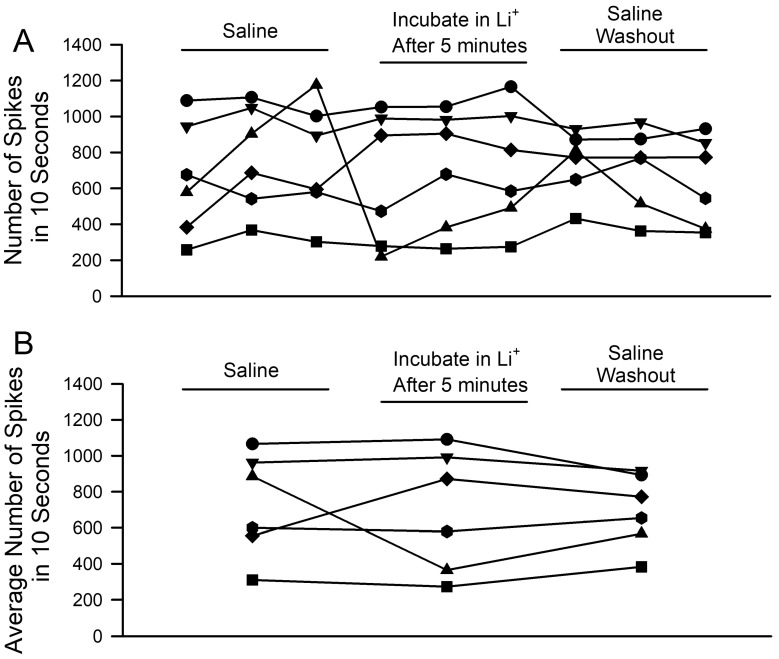
The effects of acute Na^+^ replacement by Li^+^ on PD nerve activity during joint displacement and extension. (**A**) The number of spikes within three ten-second displacements in saline, after five minutes of exposure of LiCl (470 mM- replacement of NaCl), and during a saline wash-out. Individual preparations are indicated. (**B**) The averaged activity for the three trials in each condition. (Saline to incubation in Li^+^ after 5 min; *n* = 6; paired *t*-test; *p* > 0.05).

The number of spikes recorded from the PD nerve across the three trials from each condition was initially obtained after five minutes of incubation in saline, where Na^+^ was replaced by Li^+^. Every preparation had a different activity profile and a slight variation in activity for the three trials in each condition (Figure 5A). The general trends are easier to view after the three trials’ spike counts are averaged (Figure 5B). No significant differences were noted in the overall activity for the ionic substitution (saline to incubation in Li^+^ after 5 min; *n* = 6; paired *t*-test; *p* > 0.05).

**Figure 6:**
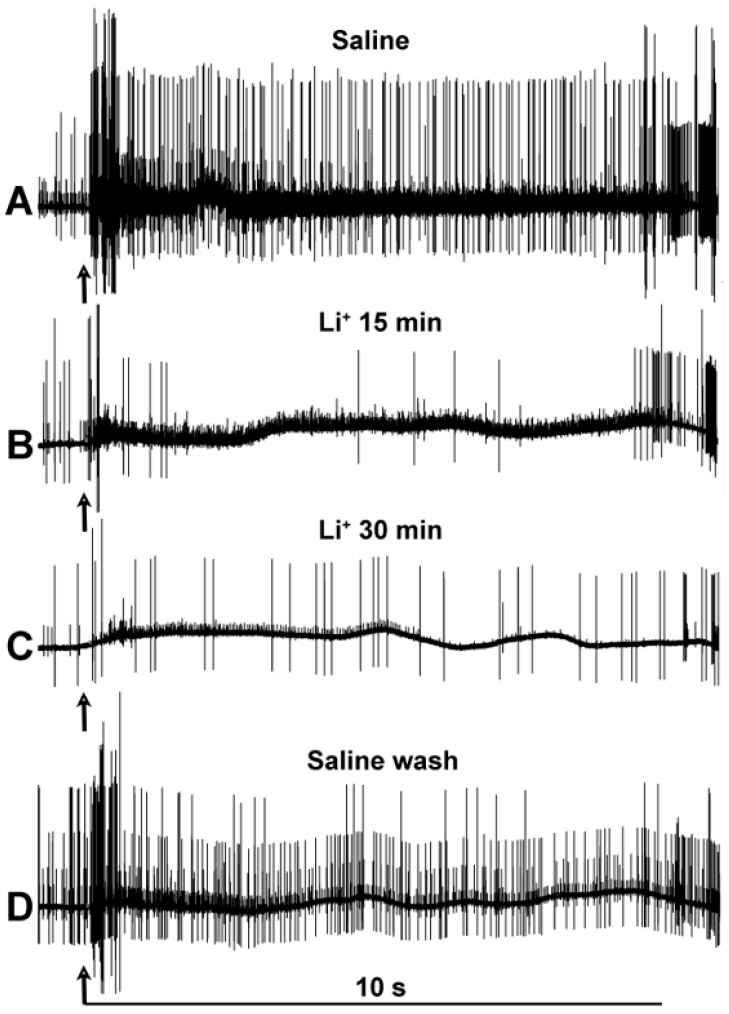
Representative activity of the PD nerve during joint displacement and extension for up to 30 min of Li^+^ substitution. (**A**) In saline with Na^+^. (**B**) After 15 min of incubation in saline with Na^+^ replaced by Li^+^. (**C**) After 30 min of incubation in saline with Na^+^ replaced by Li^+^. (**D**) After two wash-outs, flushes back to normal saline (containing Na^+^ and no Li^+^). Each trace is shown over a period of twelve seconds, illustrating the ten seconds from which analysis was conducted, one second of joint movement to an extended position, and nine more seconds of being held static before being moved back to the starting position. The arrows mark the beginning of each movement. Note that the activity prior to the joint movements also decreased with Li^+^ exposure.

Since five minutes of incubation did not reveal significant changes in the activity profiles of the PD nerve during joint displacement, a longer incubation period was utilized. The activity was assessed at both 15 and 30 min, with three trials. The activity under each condition is illustrated for one of the three trials in Figure 6.

**Figure 7:**
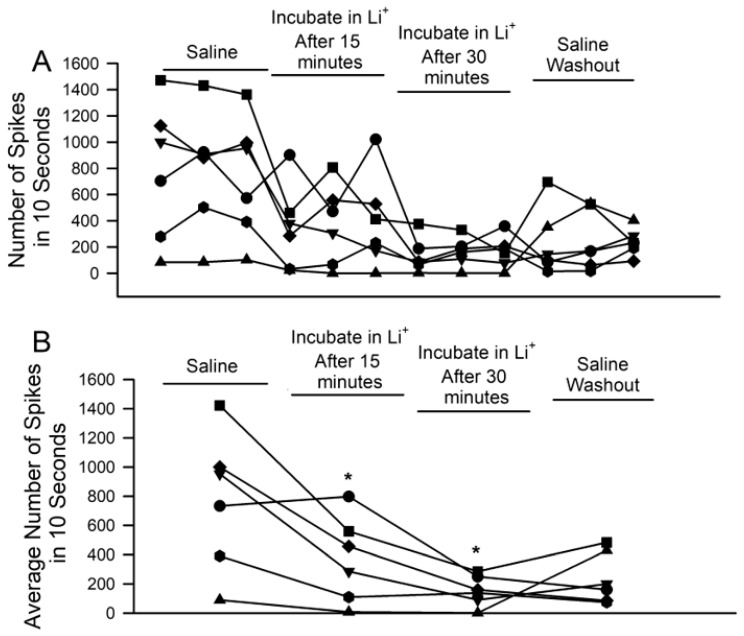
The effects of acute Li^+^ replacement of Na^+^ on PD nerve activity during joint displacement and extension for up to 30 min. (**A**) The number of spikes observed during the three ten-second displacement trials in saline, after 15 and 30 min of exposure to Li^+^ (470 mM), and during a saline wash-out. Individual preparations are indicated. (**B**) The average activity across all three trials in each condition for each preparation. (There is a significant decrease in neuronal activity after 15 or 30 min of exposure to Li^+^; *n* = 6; paired *t*-test; * *p* > 0.05).

The number of spikes observed during each of the three displacements per condition reveals how prolonged incubation in the Li^+^-environment depresses activity in each of the six preparations (Figure 7A; *n* = 6, *p* < 0.05; paired *t*-test; initial saline to 15 min or to 30 min). The average activity across each set of three trials, on the other hand, reveals the overall trends and general variation among preparations (Figure 7B). One of the six showed an average increase after 15 min, as one of its three trials saw heightened activity despite the other two substantially decreasing.

**Figure 8:**
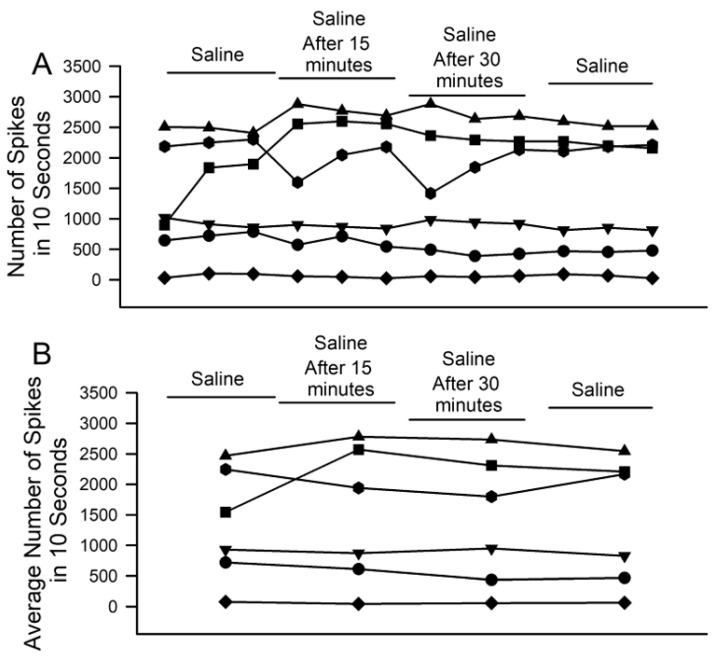
The effects of 30-min incubation time for the PD organ while exposed to saline only. (**A**) The number of spikes within the three, ten-second displacement trials in saline, after 15 and 30 min of exposure to saline only and during a saline wash-out. Individual preparations are indicated. (**B**) The average activity across the three trials in each condition for each preparation. There are no significant effects of saline exposure over time (i.e., 30 min) on neuronal activity from PD displacement (*n* = 6; paired *t*-test; *p* > 0.05).

To examine the possibility of rundown over time, control experiments with only saline over the same time period and stimulation paradigm were performed. No significant effects on neuronal activity were observed, whether from the initial saline to the 30 min of incubation or to any of the other time points examined (Figure 8; *n* = 6; paired *t*-test; *p* > 0.05).

The effects of Li^+^ on basal activity and the observed number of spikes during joint displacement are good indicators of how sensory transduction of the mechanical movement and SAC channel displacement in the sensory endings of PD neurons might function. However, to address any effects on electrical nerve conduction, the sensory transduction process was removed, and the nerve was electrically stimulated directly to monitor the effects of Li^+^ on CAP amplitude and conduction velocity. Since PD nerve activity was altered after 15 and 30 min, the same time periods were used to assess the effects on said CAPs. A representative preparation over time is illustrated with individual traces from various time points. Immediately upon bath exchange, the characteristic CAP shape changes, indicating a rapid alternation over the course of the incubation period (Figure 8).

**Figure 9:**
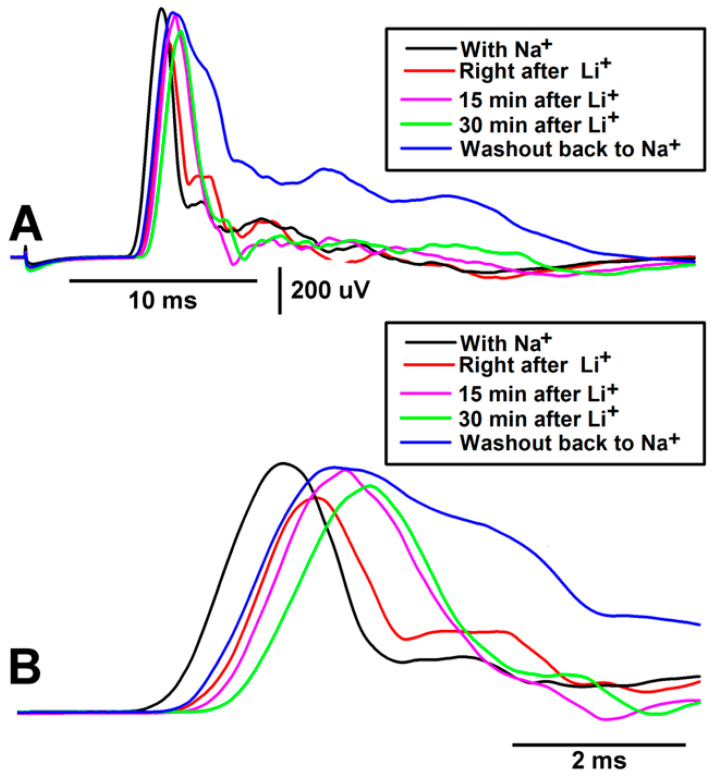
A representative preparation depicting the effects of Li^+^ replacement of Na^+^ on the leg nerve compound action potential (CAP) during evoked stimulation as superimposed traces. (**A**) Samples of CAPs before and during Li^+^ exposure at various times as well as during the return to normal saline with Na^+^, superimposed. (**B**) Enlarged images of superimposed traces are shown in A. Note: the conduction velocity slowed upon exposure to Li^+^ as well as the amplitude of the CAP peak.

To illustrate the effects of a Li^+^ environment on CAP amplitude and conduction velocity, the traces were superimposed with the stimulus artifact for reference (Figure 9A,B). Immediately after the exchange of the bathing environment, the CAPs’ amplitude and conduction speed decreased. Upon returning to the normal saline (i.e., without Li^+^), CAP amplitude and conduction speed tended to return to those observed under the original conditions. Perhaps further rinsing of the preparation with fresh saline and a longer period of wash-out exposure could obtain a full recovery; however, this was not assessed over the course of this experiment.

To summarize the effects of Li^+^ on CAP amplitude and conduction velocity (as shown in the representative preparation), the overall trends are shown in Table 1. In all six preparations, the amplitude decreased, and the conduction velocity slowed (*n* = 6; rank sum Wilcoxon test; *p* < 0.05; Initial saline to 15 min or to 30 min of exposure to Li^+^).

**Figure 10:**
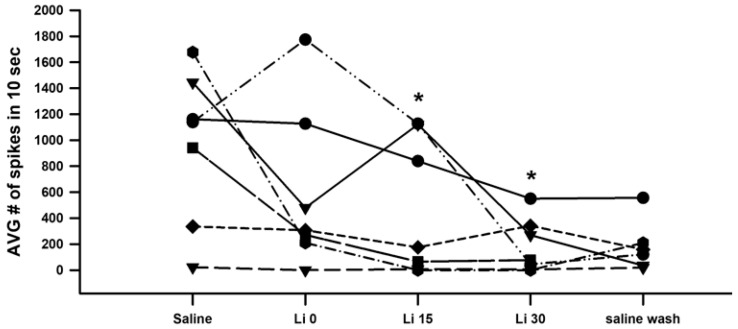
The effects of acute Li^+^ replacement of Na^+^ on PD nerve activity during joint displacement and extension, taking place over a period of time up to 30 min and in seven different recording set-ups by seven different groups of researchers. (*n* = 6; * *p* < 0.05; paired *t*-test; initial saline to 15 min or to 30 min).

In order to examine reproducibility in these observations, as well as to avoid potential bias by a given investigator, seven groups of two students each investigated the same procedures (i.e., incubation in saline where Na^+^ had been replaced by Li^+^, the same general joint movements, etc.). Seven different recording stations were run simultaneously on the same days and with the same solutions. As shown (Figure 10), the overall trends over time proved consistent with a decrease in activity during exposure to the Li^+^-containing saline (*n* = 7; *p* < 0.05; paired *t*-test). The experiments performed with these course participants may not have been as consistent, particularly in terms of the movement rate, as having only one individual compile all data sets as presented in the results above; thus, this dataset is provided separately. In each preparation, overall activity decreased after 30 min of Li^+^ exposure. However, one preparation had very little activity initially, so only a few differences in the number of spikes are not a fair representation. Thus, six preparations of the seven were better representations to consider (*n* = 6; *p* < 0.05; paired *t*-test; initial saline to 15 min or to 30 min).

In addition, four different recording set-ups, performed by ten different researchers, confirmed that the conduction velocity was reduced for the CAPs of isolated nerves in crab legs, for which the Na^+^ was replaced with Li^+^ in the bathing saline.

## 4. Discussion

This study demonstrated that Li^+^ could acutely (i.e., for up to 30 min) replace Na^+^ for electrical conduction along the sensory nerves of a marine crab, with a slight decrease in CAP amplitude and a slower electrical conduction velocity. However, the firing frequency of dynamic and static proprioceptive neurons within chordotonal organs of an intact limb joint was decreased for the ion substitution after 15 min. The proprioceptive activity was not significantly altered within only five minutes of ion substitution.

The decrease in nerve activity during joint movement and static stretching of the chordotonal organ indicates that sensory transduction within the sensory endings was compromised. The elastin and collagen within the chordotonal organ pull on the scolopendrium, housing the sensory endings [51,67,68]. The SACs within sensory ending bilipid membranes, which open and allow for ion flux sensory transduction, have yet to be pharmacologically identified, and nor has the molecular identity of the SAC protein structure been described [59,60]. Since neurons still responded to joint movement, given the absence of Ca^2+^ in the bathing medium, it would appear as though the SACs do not use Ca^2+^. Li^+^ may be close in function, as the DEG/ENaCs subtype is permeable to Na^+^ [24]. Since Li^+^ can pass through Na^+^ channels, it was surprising that nerve activity decreased during ion substitution in the bath. This finding potentially helps classify SACs by another phenomenon in addition to pharmacological profiling.

Depression of conduction velocity was induced by evoking electrical activity of the isolated nerve without sensory endings, and it is of interest to know the mechanism associated with the slowing. Since Li^+^ would be able to pass through the voltage-gated Na^+^ channels, the ion-induced current would readily be turned into current by the movement of electrons along the neurons. It is interesting that conduction velocity is slowed in this case. If Na^+^ substitution with Li^+^ altered membrane capacitance, it is possible that there would be an effect on conduction velocity. However, it is not understood why capacitance would be affected. Li^+^ has some potential effect on the K2P potassium channels responsible for the resting membrane potential and membrane leakage. If axonal input resistance was reduced, so would the amplitude of the CAPs be reduced. However, this still would not explain why the conduction velocity would be slowed.

The number of available sodium channels appears to be a factor responsible for the alteration of conduction velocity in unmyelinated axons [69]. The sodium channel inactivation following each impulse was considered to be responsible for conduction delay [69]. This would then suggest that Li^+^ influenced conduction velocity through channel inactivation and removal of inactivation. Considering that the isolated nerve was stimulated only once every five seconds and that conduction velocity slowed with Li^+^, this would imply that removal of Na^+^ channel inactivation took longer than five seconds or that Li^+^ altered the protein structure, so the rate of the conformational changes was prolonged. If the efficacy of the Na^+^-K^+^ pump is altered by Li^+^, then the membrane potential may change. Since the conduction velocity of the isolated nerve changed as soon as the medium was exchanged, the pump may be necessary to rapidly reset the potential. Future studies could address this by poisoning the pump with a blocker such as ouabain; the effect of this compound would need to be examined in this marine preparation with the intracellular recording of the axons. PD nerve axons are very small, but the neuronal cell body may offer a feasible approach. It was shown that ouabain rapidly inhibited impulse activity along the neuron associated with the MRO preparation in the freshwater crawfish [70]. Perhaps alteration of the Na^+^- (and associated K^+^-) dynamics during Li^+^ substitution is an explanation for the conduction velocity delay.

Such acute changes in neural activity through the complete replacement of Na^+^ by Li^+^ may aid understanding of lithium’s more subtle effects in health care. There does not appear to be focused research on proprioceptive function during therapeutic Li^+^ treatments. Such a focus is needed, as an alteration in sensory function, particularly of SACs in muscle spindle proprioceptors, may increase patient fall risk.

The therapeutic actions of Li^+^ treatment are not yet specifically understood [15,71]. There are various reports which state that excitatory synaptic input from glutamatergic and dopaminergic neurons is reduced and GABAergic are enhanced as a result of action on second messenger cascades [72], but it appears (in this study herein) that Li^+^ can result in reduced CAP conduction velocity and amplitude, which would result in alteration in synaptic integration timing within the CNS (central nervous system). Additionally, the reduction in recorded spike count during joint movements indicates that SAC function in sensory endings is compromised, leading to the recruitment of fewer neurons. SACs are key for mechanosensory function in mammalian proprioception, any mechanical sensory stimulus, osmolarity regulation, blood pressure control (see reviews by [23,73]), and even cell resting membrane potential, as K2P channel subtypes sense stretch and allow Na^+^ ions to pass [74,75,76]. Despite examining a high concentration of Li^+^ in these experiments, the acute results suggest that, in other organisms, it may potentially have subtle effects on SACs, neuronal excitability, and electrical conduction, given longer periods of lower-concentration exposure.

The data presented within this paper were reliably reproduced in the physiological recordings and data analysis of participants within a neurophysiology course. Eighteen students, working in groups of two, conducted the same set of experiments with the same protocols, altering the concentration of LiCl. While the classroom lacked some of the features of a controlled lab, such as vibration-free tables, and while the students may have mobilized the joint at different rates, the same general trends in data were found; specifically, the number of spikes decreased in the observed preparations. This model of classroom-based testing followed the ACURE (authentic course-based undergraduate research experiences) method, building off of the CURE (course-based undergraduate research experiences) concept. Using this ACURE model, students were immersed in an authentic research experience that not only aided in learning the concepts being presented but also exposed them to the communication, trial-and-error, and teamwork components of research.

Future research is needed to address the molecular, pharmacological, and physiological SAC subtypes in insect and crustacean chordotonal organs. Additionally, the description of Li^+^ as an ion for flux through SACs would be of use for understanding the mechanism of action behind altered proprioceptive activity. With patch clamp recordings, it would be possible to identify the effect of Li^+^ on voltage-gated Na^+^ channel inactivation and removal of inactivation. Intracellular recordings would also assist in determining the effects on membrane potential, as well as addressing the mechanism behind slowed condition velocity in neurons.

## Figures and Tables

**Table 1:** The effect of Li^+^ on compound action potential (CAP) and conduction velocity.

Preparation	Amplitude of CAP	Conduction Velocity	Trend to Recover in Saline Rinse
1	↓	↓	+
2	↓	↓	+
3	↓	↓	+
4	↓	↓	+
5	↓	↓	+
6	↓	↓	+

## Data Availability

Most of all raw data are presented in this manuscript. Other data are available upon request.

## References

[1] Janka Z., Jones D.G. (1982). Lithium entry into neural cells via sodium channels: A morphometric approach. Neuroscience.

[2] Obara S., Grundfest H. (1968). Effects of lithium on different membrane components of crayfish stretch receptor neurons. J. Gen. Physiol..

[3] Baldessarini R.J., Tondo L., Vázquez G.H. (2019). Pharmacological treatment of adult bipolar disorder. Mol. Psychiatry.

[4] Fountoulakis K.N., Tohen M., Zarate C.A. (2022). Lithium treatment of Bipolar disorder in adults: A systematic review of randomized trials and meta-analyses. Eur. Neuropsychopharmacol..

[5] Baird-Gunning J., Lea-Henry T., Hoegberg L.C.G., Gosselin S., Roberts D.M. (2017). Lithium poisoning. J. Intensive Care Med..

[6] Mifsud S., Cilia K., Mifsud E.L., Gruppetta M. (2020). Lithium-associated hyperparathyroidism. Br. J. Hosp. Med..

[7] Kato M., Lledo P.M., Vincent J.D. (1991). Blockade by lithium ions of potassium channels in rat anterior pituitary cells. Am. J. Physiol..

[8] Carrasquillo Y., Nerbonne J.M. (2014). IA channels: Diverse regulatory mechanisms. Neuroscientist.

[9] Thomas L., Xue J., Dominguez Rieg J.A., Rieg T. (2019). Contribution of NHE3 and dietary phosphate to lithium pharmacokinetics. Eur. J. Pharm. Sci..

[10] Banerjee U., Dasgupta A., Rout J.K., Singh O.P. (2012). Effects of lithium therapy on Na^+^-K+-ATPase activity and lipid peroxidation in bipolar disorder. Prog. Neuropsychopharmacol. Biol. Psychiatry.

[11] Fleĭshman D.G. (1991). Li^+^ as a Na^+^ analog in ion transport process in vertebrates. Tsitologiia.

[12] Dudev T., Mazmanian K., Lim C. (2018). Competition between Li^+^ and Na^+^ in sodium transporters and receptors: Which Na^+^-Binding sites are “therapeutic” Li^+^ targets?. Chem. Sci..

[13] Oliveira J.L., Silva Júnior G.B., Abreu K.L., Rocha Nde A., Franco L.F., Araújo S.M., Daher Ede F. (2010). Lithium nephrotoxicity. Rev. Assoc. Med. Bras..

[14] Gitlin M. (2016). Lithium side effects and toxicity: Prevalence and management strategies. Int. J. Bipolar Disord..

[15] Morris G., Berk M. (2016). The putative use of lithium in Alzheimer’s disease. Curr. Alzheimer Res..

[16] Nyirenda M.J., Tang J.I., Padfield P.L., Seckl J.R. (2009). Hyperkalaemia. Brit. Med. J..

[17] Hodgkin A.L., Horowicz P. (1959). The influence of potassium and chloride ions on the membrane potential of single muscle fibres. J. Physiol..

[18] Kristensen S.R. (1994). Mechanisms of cell damage and enzyme release. Danish Med. Bull..

[19] Orkand R.K., Nicholls J.G., Kuffler S.W. (1966). Effect of nerve impulses on the membrane potential of glial cells in the central nervous system of amphibia. J. Neurophysiol..

[20] Grafe P., Reddy M.M., Emmert H., ten Bruggencate G. (1983). Effects of lithium on electrical activity and potassium ion distribution in the vertebrate central nervous system. Brain Res..

[21] Coste B., Mathur J., Schmidt M., Earley T.J., Ranade S., Petrus M.J., Dubin A.E., Patapoutian A. (2010). Piezo1 and Piezo2 are essential components of distinct mechanically activated cation channels. Science.

[22] Arnadóttir J., Chalfie M. (2010). Eukaryotic mechanosensitive channels. Annu. Rev. Biophys..

[23] Sachs F. (2010). Stretch-activated ion channels: What are they?. Physiology.

[24] Geffeney S.L., Goodman M.B. (2012). How we feel: Ion channel partnerships that detect mechanical inputs and give rise to ouch and pain perception. Neuron.

[25] Richet C. (1879). Contributions à la physiologie des centres nerveux et des muscles de l’écrevisse. Arch. Physiol. Norm. Path..

[26] Richet C., Paris G. (1882). (Physiologie des Muscles Et des Nerfs: Leçons Professées à la Faculté de Médecine en 1881) par Charles Richet.

[27] Huxley T.H., Paul C.K. (1880). The Crayfish an Introduction to the Study of Zoology.

[28] Wiersma C.A.G. (1993). Vergleichende Untersuchungen über das periphere Nerve-muskel-system von Crustaceen. Zeitschr. vergl. Physiol..

[29] Van Harreveld A. (1936). A physiological solution for freshwater crustaceans. Proc. Soc. Exp. Biol. Med..

[30] Prosser C.L. (1940). Effects of salts upon “spontaneous” activity in the nervous system of the crayfish. J. Cell. Comp. Physiol..

[31] Hodgkin A.L., Rushton W.A.H. (1946). The electrical constants of a crustacean nerve fibre. Proc. Roy. Soc..

[32] Fatt P., Katz B. (1953). Distributed ‘endplate potentials’ of crustacean muscle fibres. J. Exp. Biol..

[33] Skou J.C. (1965). Enzymatic basis for active transport of Na^+^ and K+ across cell membrane. Physiol. Rev..

[34] Skou J.C. (1998). Nobel Lecture. The identification of the sodium pump. Biosci Rep..

[35] Robbins J. (1959). The excitation and inhibition of crustacean muscle by amino acids. J. Physiol..

[36] Furshpan E.J., Potter D.D. (1959). Transmission at the giant motor synapses of the crayfish. J. Physiol..

[37] Wiersma C.A.G., Hughes G.M. (1961). On the functional anatomy of neuronal units in the abdominal cord of the crayfish, *Procambarus clarkii*. J. Comp. Neurol..

[38] Dudel J., Kuffler S.W. (1961). Presynaptic inhibition at the crayfish neuromuscular junction. J. Physiol..

[39] Dudel J., Kuffler S.W. (1961). The quantal nature of transmission and spontaneous miniature potentials at the crayfish neuromuscular junction. J. Physiol..

[40] Alexandrowicz J.S. (1951). Muscle receptor organs in the abdomen of Homarus vulgaris and Palinurus vulgaris. Q. J. Microsc. Sci..

[41] Kuffler S.W. (1954). Mechanisms of activation and motor control of stretch receptors in lobster and crayfish. J. Neurophysiol..

[42] Eckert R.O. (1961). Reflex relationships of the abdominal stretch receptors of the crayfish. I. Feedback inhibition of the receptors. J. Cell. Comp. Physiol..

[43] Eckert R.O. (1961). Reflex relationships of the abdominal stretch receptors of the crayfish. II. Stretch receptor involvement during the swimming reflex. J. Cell. Comp. Physiol..

[44] Rydqvist B., Purali N. (1991). Potential-dependent potassium currents in the rapidly adapting stretch receptor neuron of the crayfish. Acta Physiol. Scand..

[45] Rydqvist B., Swerup C. (1991). Stimulus-response properties of the slowly adapting stretch receptor neuron of the crayfish. Acta Physiol. Scand..

[46] Bullock T., Horridge G.A. (1965). Structure and Function in the Nervous Systems of Invertebrates.

[47] Mill P.J. (1976). Chordotonal organs of crustacean appendages. Structure and Function of Proprioceptors in the Invertebrates.

[48] Alexandrowicz J.S. (1958). Further observations on proprioceptors in Crustacea and a hypothesis about their function. J. Mar. Biol. Assoc. UK.

[49] Alexandrowicz J.S. (1967). Receptor organs in the coxal region of Palinurus vulgaris. J. Mar. Biol. Assoc. UK.

[50] Alexandrowicz J.S. (1972). The comparative anatomy of leg proprioceptors in some decapod Crustacea. J. Mar. Biol. Assoc. UK.

[51] Whitear M. (1960). Chordotonal organs in Crustacea. Nature.

[52] Bush B.M.H. (1962). Proprioceptive reflexes in the legs of Carcinus meanas. J. Exp. Biol..

[53] Bush B.M.H. (1965). Proprioception by chordotonal organs in the mero-carpopodite and carpo-propodite joints of Carcinus maenas legs. Comp. Biochem. Physiol..

[54] Bush B.M.H. (1965). Proprioception by the coxo-basal chordotonal organ, CB, in legs of the crab, Carcinus maenas. J. Exp. Biol..

[55] Cooper R.L. (2008). Proprioceptive neurons of chordotonal organs in the crab, *Cancer magister* dana (Decapoda, Brachyura). Crustaceana.

[56] Cooper R.L., Hartman H.B. (1999). Quantification of responses from proprioceptive neurons in the limbs of the crab, Cancer magister. J. Exp. Zool..

[57] Hartman H.B., Boettiger E.G. (1967). The functional organization of the propus-dactylus organ in Cancer irroratus Say. Comp. Biochem. Physiol..

[58] Atkins D.E., Bosh K.L., Breakfield G.W., Daniels S.E., Devore M.J., Fite H.E., Guo L.Z., Henry D.K.J., Kaffenberger A.K., Manning K.S. (2021). The Effect of Calcium Ions on Mechanosensation and Neuronal Activity in Proprioceptive Neurons. NeuroSci.

[59] Dayaram V., Malloy C., Martha S., Alvarez B., Chukwudolue I., Dabbain N., Dmahmood D., Goleva S., Hickey T., Ho A. (2017). The effect of CO_2_, intracellular pH and extracellular pH on mechanosensory proprioceptor responses in crayfish and crab. Am. J. Undergrad. Res..

[60] McCubbin S., Jeoung A., Waterbury C., Cooper R.L. (2020). Pharmacological profiling of stretch activated channels in proprioceptive neuron. Comp. Biochem. Physiol. C.

[61] Stanley C.E., Adams R., Nadolski J., Amrit E., Barrett M., Bohnett C., Campbell K., Deweese K., Dhar S., Gillis B. (2020). The effects of tricaine mesylate on arthropods: Crayfish, crab and Drosophila. Invertebr. Neurosci..

[62] Pankau C., Nadolski J., Tanner H., Cryer C., Di Girolamo J., Haddad C., Lanning M., Miller M., Neely D., Wilson R. (2022). Examining the effect of manganese on physiological processes: Invertebrate models. Comp. Biochem. Physiol. C Toxicol. Pharmacol..

[63] Tanner H.N., Atkins D.E., Bosh K.L., Breakfield G.W., Daniels S.E., Devore M.J., Fite H.E., Guo L.Z., Henry D.K.J., Kaffenberger A.K. (2022). Effect of TEA and 4-AP on primary sensory neurons in a crustacean model. J. Pharmacol. Toxicol..

[64] Ison B.J., Abul-Khoudoud M.O., Ahmed S., Alhamdani A.W., Ashley C., Bidros P.C., Bledsoe C.O., Bolton K.E., Capili J.G., Henning J.N. (2022). The effect of doxapram on proprioceptive neurons: Invertebrate model. NeuroSci.

[65] O’Neil A.S., Krall R.M., Vascassenno R., Cooper R.L. (2023). Exploring mechanisms in a medical treatment for a disease: A teaching/learning module. Advances in Biology Laboratory Education. Publ. Assoc. Biol. Lab. Educ. ABLE.

[66] Majeed Z.R., Titlow J., Hartman H.B., Cooper R.L. (2013). Proprioception and tension receptors in crab limbs: Student laboratory exercises. J. Vis. Exp..

[67] Whitear M. (1962). The fine structure of crustacean proprioceptors. I. The chordotonal organs in the legs of the shore crab, Carcinus meanas. Phil. Trans. Roy. Soc. Lond. B.

[68] Whitear M. (1965). The fine structure of crustacean proprioceptors. II. The thoracico-coxal organs in Carcinus, Pagurus and Astacus. Phil. Trans. Roy. Soc. Lond. B.

[69] De Col R., Messlinger K., Carr R.W. (2008). Conduction velocity is regulated by sodium channel inactivation in unmyelinated axons innervating the rat cranial meninges. J. Physiol..

[70] Giacobini E. (1966). The effect of metabolic and ion transport inhibitors on the impulse activity and oxygen uptake of an isolated crustacean neurone. Acta Physiol. Scand..

[71] Rybakowski J. (2020). Lithium treatment—The state of the art for 2020. Psychiatr. Pol..

[72] Malhi G.S., Tanious M., Das P., Coulston C.M., Berk M. (2013). Potential mechanisms of action of lithium in bipolar disorder. Current understanding. Current understanding. CNS Drugs..

[73] Richardson J., Kotevski A., Poole K. (2022). From stretch to deflection: The importance of context in the activation of mammalian, mechanically activated ion channels. FEBS J..

[74] Kamuene J.M., Xu Y., Plant L.D. (2021). The pharmacology of two-pore domain potassium channels. Handbook of Experimental Pharmacology.

[75] Sepúlveda F.V., Pablo Cid L., Teulon J. (2015). Niemeyer MI. Molecular aspects of structure, gating, and physiology of pH-sensitive background K2P and Kir+-transport channels. Physiol. Rev..

[76] Mita K., Sumikama T., Iwamoto M., Matsuki Y., Shigemi K., Oiki S. (2021). Conductance selectivity of Na^+^ across the K^+^ channel via Na^+^ trapped in a tortuous trajectory. Proc. Natl. Acad. Sci. USA.

